# siRNA that participates in *Drosophila* dosage compensation is produced by many 1.688^X^ and 359 bp repeats

**DOI:** 10.1093/genetics/iyae074

**Published:** 2024-05-08

**Authors:** Sudeshna Biswas, Katherine Gurdziel, Victoria H Meller

**Affiliations:** Department of Biological Sciences, Wayne State University, 5047 Gullen Mall, Detroit, MI 48202, USA; Department of Pharmacology, Wayne State University, Integrative Bioscience Center (iBio), 6135 Woodward, Detroit, MI 48202, USA; Institute of Environmental Health Sciences, Wayne State University, Integrative Bioscience Center (iBio), 6135 Woodward, Detroit, MI 48202, USA; Department of Biological Sciences, Wayne State University, 5047 Gullen Mall, Detroit, MI 48202, USA

**Keywords:** dosage compensation, X recognition, *Drosophila melanogaster;* siRNA, small RNA, 1.688^X^ satellite repeats, *roX*, 359 bp repeats, epigenetics

## Abstract

Organisms with differentiated sex chromosomes must accommodate unequal gene dosage in males and females. Male fruit flies increase X-linked gene expression to compensate for hemizygosity of their single X chromosome. Full compensation requires localization of the Male-Specific Lethal (MSL) complex to active genes on the male X, where it modulates chromatin to elevate expression. The mechanisms that identify X chromatin are poorly understood. The euchromatic X is enriched for AT-rich, ∼359 bp satellites termed the 1.688^X^ repeats. Autosomal insertions of 1.688^X^ DNA enable MSL recruitment to nearby genes. Ectopic expression of dsRNA from one of these repeats produces siRNA and partially restores X-localization of MSLs in males with defective X recognition. Surprisingly, expression of double-stranded RNA from three other 1.688^X^ repeats failed to rescue males. We reconstructed dsRNA-expressing transgenes with sequence from two of these repeats and identified phasing of repeat DNA, rather than sequence or orientation, as the factor that determines rescue of males with defective X recognition. Small RNA sequencing revealed that siRNA was produced in flies with a transgene that rescues, but not in those carrying a transgene with the same repeat but different phasing. We demonstrate that pericentromeric X heterochromatin promotes X recognition through a maternal effect, potentially mediated by small RNA from closely related heterochromatic repeats. This suggests that the sources of siRNAs promoting X recognition are highly redundant. We propose that enrichment of satellite repeats on Drosophilid X chromosomes facilitates the rapid evolution of differentiated sex chromosomes by marking the X for compensation.

## Introduction

Many species have evolved highly differentiated X and Y chromosomes that create an imbalance in gene dosage between males and females (Disteche 2012). To ensure equivalent expression of X-linked genes, these species have evolved a process termed dosage compensation. Mammals achieve dosage compensation by inactivating one of the two X chromosomes in females, but *Drosophila* employ a different strategy (Charlesworth 1996; Lucchesi *et al.* 2005). Fruit fly males upregulate expression from their single X chromosome about two-fold (Lucchesi 1978). A ribonucleoprotein complex called the Male-Specific Lethal complex or Dosage Compensation Complex (MSL or DCC) is necessary for full dosage compensation in male flies (Gelbart and Kuroda 2009). The MSL complex is made up of five proteins, Male-Specific Lethal 1, -2 and -3 (MSL1; MSL2; and MSL3); Maleless (MLE) and Males absent On the First (MOF). MOF, a histone acetyltransferase, acetylates histone H4 at lysine 16 (H4K16Ac) (Hilfiker *et al.* 1997; Smith *et al.* 2000). Acetylation at this position is associated with active genes and decondensed chromatin (Grunstein 1997; Shogren-Knaak *et al.* 2006). H4K16 is highly enriched over the body of X-linked genes and this mark is thought to be responsible for transcriptional upregulation in males (Bone *et al.* 1994; Hilfiker *et al.* 1997; Akhtar and Becker 2000; Smith *et al.* 2000; Copur *et al.* 2018). In addition to these five proteins, one of two functionally redundant long noncoding RNAs, *RNA on the X 1* and -*2* (*roX1* and *roX2*), also participate in MSL complex formation (Amrein and Axel 1997; Meller *et al.* 1997; Ilik *et al.* 2013). Simultaneous mutation of both *roX* genes disrupts X-localization of MSL proteins and leads to male lethality (Meller and Rattner 2002; Deng *et al.* 2005; Deng and Meller 2006).

Although the components of the dosage compensation complex and their functions are well studied, how the MSL complex selectively recognizes the X chromosome is not clear. One model suggests that the MSL complex forms on *roX* transcripts and is recruited to 200–300 X-linked Chromatin Entry Sites, also termed High Affinity Sites (CES, HAS) (Kelley *et al.* 1999; Park *et al.* 2002; Oh *et al.* 2003). CES are enriched for a 21 bp GA-rich MRE (MSL Recognition Element) motif (Alekseyenko *et al.* 2008). A zinc finger protein called Chromatin-Linked Adaptor for MSL Proteins (CLAMP), binds MRE sequences throughout the genome, but selectively recruits the MSL complex to X-linked CES (Soruco *et al.* 2013). The MSL complex then spreads to nearby genes through binding of MSL3 to the co-transcriptional H3K36me3 mark (Bell *et al.* 2007; Kind and Akhtar 2007; Larschan *et al*. 2007; Sural *et al.* 2008). Although autosomal MRE motifs bind CLAMP, they do not recruit the MSL complex. A CES variant termed PionX (Pioneering sites on the X) is reported to interact with CLAMP and a domain of MSL2 (Villa *et al.* 2016). These sites are among the first CES to be bound during the assembly of MS complex and are enriched on the X chromosome, suggesting a role in distinguishing the X chromosome and autosomes (Albig *et al.* 2019).

Both *roX* genes are X-linked and overlap CES. *roX* transgenes recruit compensation to nearby active genes when inserted on an autosome, suggesting a role in marking the X chromosome (Kelley *et al.* 1999; Henry *et al.* 2001; Joshi and Meller 2017). However, *roX* RNA produced from an autosomal transgene is incorporated into the MSL complex and travels to the X chromosome to rescue compensation in *roX1 roX2* males (Meller and Rattner 2002). This indicates that *roX* genes are not sufficient to confer X-identity. A chromosome conformation study demonstrated that CES are spatially arranged close to each other in the male interphase nucleus (Ramírez *et al.* 2015). Long-range interactions near active X-linked genes are more pronounced in males compared to females, which suggests that chromosome organization contributes to X recognition and dosage compensation (Grimaud and Becker 2009; Pal *et al.* 2019).

Previous studies from our lab have established a role for small interfering RNA (siRNA) in X recognition (Menon and Meller 2012; Menon *et al.* 2014). There are hundreds 1.688^X^ satellite repeats (CsCl density of 1.688 g/cm^3^) in short, tandem repeats in euchromatin of the *Drosophila* X chromosome (Hsieh and Brutlag 1979; Waring and Pollack 1987; DiBartolomeis *et al.* 1992; Kuhn *et al.* 2012). These are AT-rich, have a repeat unit of ∼359 bp and are dissimilar to CES. Many 1.688^X^ repeats are transcribed and small RNA aligning to 1.688^X^ repeats is present in early embryos and the maternal germ line, but rare or absent in later development (Menon *et al.* 2014). Ectopic expression of double-stranded RNA (dsRNA) from a repeat at cytological position 3F (1.688^3F^) dramatically rescued the survival of *roX1 roX2* males and partially restored X-localization of the MSL proteins (Menon *et al.* 2014). Sequencing revealed substantial production of siRNAs upon 1.688^3F^ dsRNA expression. In addition, autosomal transgenes containing 1–2 kb of DNA from 1.688^3F^, as well as repeats at 1A and 3C (1.688^1A^, 1.688^3C^) recruit MSL proteins in males and increase expression of genes up to 150 kb away (Joshi and Meller 2017; Deshpande and Meller 2018). This suggests a role for 1.688^X^ DNA in marking the X, and for small RNA from these repeats in the process of X recognition.

Repeats at 1.688^1A^, 1.688^3C^, and 1.688^4A^ were selected to create dsRNA-expressing transgenes as these repeats range from very similar to quite divergent from 1.688^3F^ (89, 67 and 95% identity, respectively). Surprisingly, dsRNA-producing transgenes constructed with 1.688^1A^, 1.688^3C^, and 1.688^4A^ sequences failed to rescue *roX1 roX2* males (Menon *et al.* 2014, and unpublished). This was baffling because insertion of 1.688^1A^ or 1.688^3C^ DNA on an autosome attracts the compensation machinery to nearby genes, indicating that these repeats do contribute to marking the X for compensation (Joshi and Meller 2017). Intriguingly, 1.688^3F^ is immediately distal to *roX1.* The *Drosophila* genome annotation depicts a *roX1* transcript extending through the 1.688^3F^ repeat, but we have been unable to detect this extended transcript by amplification of cDNA and believe that virtually all *roX1* transcripts end at termination signals between *roX1* and the 1.688^3F^ repeats. Nevertheless, the proximity of these elements is provocative and suggested that the 1.688^3F^ repeat might have a unique biological function. Notably, *roX1* is expressed 1.5 h after egg laying (AEL) and supports initial X recognition (Meller 2003). In *roX1* mutant males, X-localization of the MSL proteins is delayed until the onset of *roX2* expression at 6 h AEL. These observations suggested that two RNA-producing elements at 3F might collaborate to drive initial X recognition.

It is possible that only siRNA from 1.688^3F^ is capable of promoting X recognition and achieving rescue of *roX1 roX2* males, and that sequence differences between the 1.688^X^ repeats previously tested are critical. Alternatively, details of construction of the transgenes expressing dsRNA from each repeat could determine function. To address these questions, we designed studies to determine if repeat sequence determines biological activity, or if the details of transgene construction play a crucial role.

We show that dsRNA from the 1.688^1A^ and 1.688^3C^ repeats rescues *roX1 roX2* males when the expressing transgenes are constructed with the same repeat phasing as used to generate the original 1.688^3F^ dsRNA construct. Selection of these repeats enables direct comparisons between the original 1.688^1A^ and 1.688^3C^ dsRNA transgenes that fail to rescue *roX1 roX2* males and reconstructed versions containing 1.688^1A^ and 1.688^3C^ sequences. Small RNA sequencing revealed that 1.688^1A^ dsRNA is processed into small RNA when the phasing of the repeat matches that of the original 1.688^3F^ dsRNA construct. In contrast, virtually no siRNA aligning to 1.688^1A^ was detected in flies expressing dsRNA 1.688^1A^ from the original transgene that does not rescue *roX1 roX2* males. We conclude that the distinction between the repeats previously tested is due to details of transgene construction, not DNA sequence. This suggests that many of the 1.688^X^ repeats on the X are capable of producing small RNA promoting X recognition. We provide evidence that the closely related pericentromeric 359 bp repeats, comprising 10 Mb of X heterochromatin, as well as shorter blocks on the second and third chromosomes, act maternally to promote X recognition, possibly through production of siRNAs that are transmitted to the zygote. These findings indicate high redundancy in the sources of satellite siRNAs that promote X recognition.

## Materials and methods

### Fly culture and genetics

Flies were maintained at room temperature on yeast and molasses media. Strains used in these studies are presented in Supplementary Table 1. Transgenes were injected by Rainbow Transgenics (Camarillo, CA). Three independent second chromosome insertions of each construct were selected for further studies. Integrations of *roX1* and 1.688^3F^ recruiting elements in the *haf* gene were previously described (Joshi and Meller 2017). Integrations were recombined with p[w^+^Sqh-Gal4]2 and homozygous adults mated to flies carrying reconstructed pWIZ transgenes. Groups of 60 larvae were collected for RNA isolation and RT-qPCR (primers in Supplementary Table 3). *roX1^ex33A^ roX2Δ Zhr^1^* recombinants were identified by single fly qPCR.

### Generation of reconstructed transgenes

All dsRNA-expressing constructs were created using the pWIZ (White Intron Zipper) vector (Lee and Carthew 2003). Reconstructed repeats with the same size and repeat phasing as the original pWIZ-ds1.688^3F^ transgene are designated by a superscript indicating the repeat followed by R (1.688^1AR^, 1.688^3CR^). Due to high AT content, the 1.688^1AR^ repeat was synthesized as two gene blocks based on sequence downloaded from Flybase (FB2020; Öztürk-Çolak *et al.* 2024), synthesized by Integrated DNA Technologies and joined at a naturally occurring *EcoR*I site. Blocks were amplified, digested with *EcoR*I, ligated, digested with *Xba*I, gel-purified, ligated into pBlueScript, and confirmed by sequencing. Primers used for the generation and validation of transgenes are presented in Supplementary Table 2. Assembly of 1.688^3CR^ was accomplished by PCR of overlapping fragments templated with an existing 901 bp 1.688^3C^ amplicon (Menon *et al.* 2014). Gel-purified amplicons were mixed and subjected to five cycles of denaturation, annealing, and extension. This templated an amplification with flanking primers 3C_Fo and 3C_Ro. A product of the correct size was gel purified, digested with *Xho1* and *Not1*, inserted into pBlueScript and confirmed by sequencing. 1.688^1AR^ and 1.688^3CR^ inserts were introduced sequentially into pWIZ and orientation was verified by sequencing after each step. Random insertions were mapped to chromosome and three independent insertions of teach transgene selected for further studies.

### Rescue of *roX1 roX2* males

Males homozygous for each reconstructed transgene were mated to *roX1 roX2*; p[*w^+^*Sqh-Gal4]2/+ virgins to produce offspring with and without the p[*w^+^*Sqh-Gal4]2 driver. Offspring were counted for 10 days following initial eclosion and eye color was used to distinguish flies with the p[*w^+^*Sqh-Gal4]2 driver. The driver had no effect on female survival. For this reason, the total number of daughters was used to calculate survival of each class of sons (Supplementary Tables 4 and 5).

### RNA preparation

Groups of 60 third instar male larvae were homogenized in Trizol (Invitrogen). RNA was cleaned using the QIAGEN RNeasy kit and measured by nanodrop as previously described (Koya and Meller 2015). One μg of RNA was reverse transcribed using random hexamers and Invitrogen SuperScript IV reverse transcriptase.

### Quantitative RT-PCR

Amplification of cDNA was performed using Power SYBR Green PCR master mix on a Quantstudio3 qPCR system (ThermoFisher Scientific). Primers are presented in Supplementary Table 3. Values were normalized to *dmn* (DCTN2-p50). Fold change in expression was calculated relative to the *yw* lab reference strain using the efficiency corrected comparative quantification method (Pfaffl 2001). The high copy number of pericentric 359 bp repeats required serial dilution of qPCR template. To determine the influence of dsRNA expression on an autosomal gene with integrated recruiting elements, transgene insertions that achieved the strongest rescue of *roX1 roX2* males were selected from the three independent insertions tested.

### siRNA sequencing and alignment

Total RNA prepared as described above was shipped to Azenta Life Sciences for siRNA isolation and sequencing. Libraries were generated using the NEB Small RNA library Prep Kit (Ipswich, MA, USA) and Illumina 3′ and 5′ adapters. Index sequences were added by amplification and products of 145–160 bp were gel purified and sequenced on an Illumina NovaSeq (2 × 150 bp). Raw sequencing reads underwent quality analyses and adapter trimming using Trimmomatic (v0.30). Trimmed reads of 18 to 30 bp were aligned to the *Drosophila* genome (dm6) with bowtie2 with and default parameters (Hoskins *et al*. 2015). Reads were also aligned to the transcribed region of pWIZ-1.688^1AR-inv^ using bowtie 1 (-l 10 -m 100) with zero mismatches.

## Results

### Details of transgene construction determine activity

**Fig. 1.**
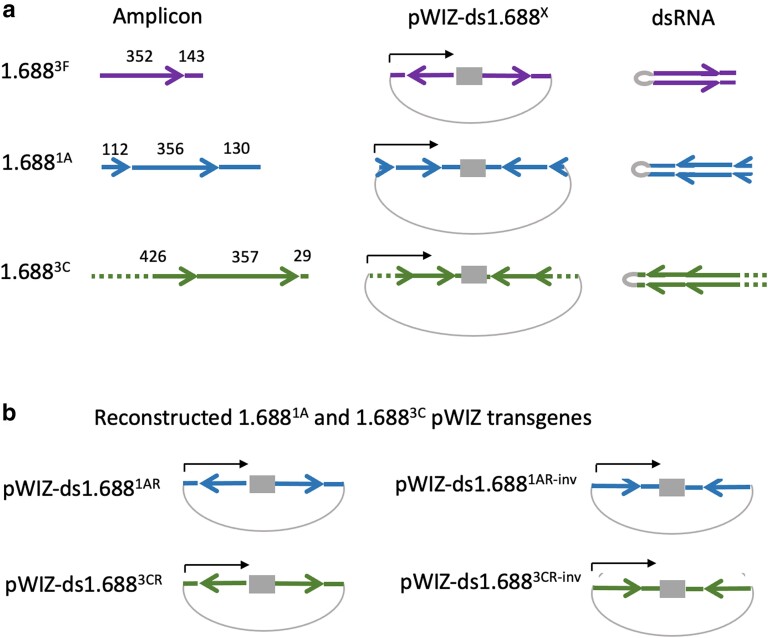
Original and reconstructed 1.688^X^ pWIZ transgenes. a) Left—1.688^3F^, 1.688^1A^, and 1.688^3C^ amplicons used in original pWIZ-ds1.688^X^ transgenes (Menon *et al.* 2014). Arrows represent ∼359 bp repeat units. *Eco*R1 sites present in many 1.688^X^ repeats are arbitrarily designated as the endpoint of each repeat unit. The dotted line in 1.688^3C^ represents a region with limited repeat homology. Center—orientation of inserts in original constructs. Right—dsRNAs produced by each construct. b) Inserts in reconstructed pWIZ-ds1.688^1AR^ and pWIZ-ds1.688^3CR^ are the same size, orientation, and phasing as the original pWIZ-ds1.688^3F^. The orientation of inserts in pWIZ-ds1.688^1AR-inv^ and pWIZ-ds1.688^3CR-inv^ is inverted.

**Fig. 2.**
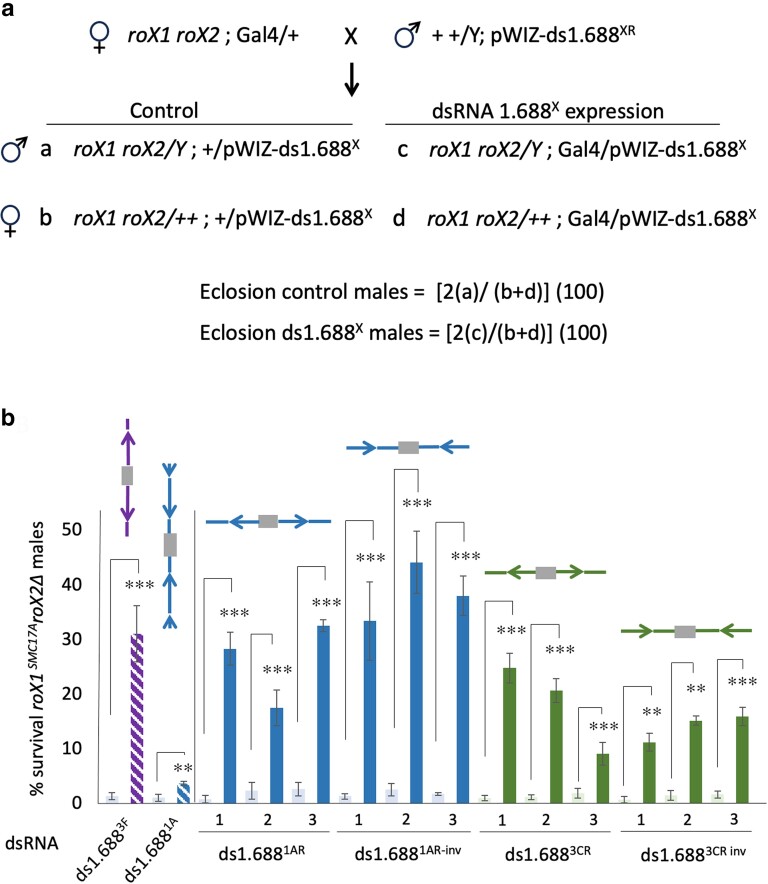
Expression of reconstructed ds1.688^X^ transgenes rescues *roX1^SMC17A^ roX2*Δ males. a) *roX1^SMC17A^ roX2Δ* females heterozygous for the p[w^+^Sqh-GAL4]2 driver were mated to males carrying pWIZ transgenes. The recovery of male offspring was normalized by multiplying males of each category by two and dividing by the total number of daughters. b) The original pWIZ-ds1.688^3F^ and pWIZ-ds1.688^1A^ (hatched bars) and three independent insertions of each reconstructed transgenic (dark bars) were tested. The survival of males with each pWIZ transgene but without driver is shown by light gray bars. Error bars represent the SEM of four biological replicates. *P < 0.05; **P < 0.01, ***P < 0.001, as determined by Student’s *t* test.

The idea that the 1.688^3F^ repeat, situated immediately distal to *roX1,* had special properties was intriguing. However, details of construction differed for the original pWIZ-ds1.688^1A^, pWIZ-ds1.688^3C^, and pWIZ-ds1.688^3F^ transgenes (Menon *et al*. 2014). The amplified repeat segments were of different sizes (Fig. 1a and Supplementary Fig. 3). The ends of each amplicon also fell at different places in the 359 bp repeat unit, and thus each amplicon differed in repeat phasing. Many of the 1.688^X^ repeats contain an *EcoR*I site that we arbitrarily designated as the boundary of individual repeats in tandem clusters. A two base pair change in 1.688^3C^ converts *EcoR*I to *Sac*I (see Supplementary Fig. 3). Finally, the orientation of repeat DNA introduced into pWIZ differed between pWIZ-ds1.688^3F^ and the other two original constructs (Fig. 1a). We first performed PCR and sequencing on flies with the original pWIZ-ds1.688^1A^ and pWIZ-ds1.688^3C^ transgenes and confirmed that these were constructed as intended and had not been rearranged in transgenic flies. Reverse transcription and qPCR (RT-qPCR) of RNA from flies carrying pWIZ-ds1.688^1A^ and the strong, near-ubiquitous p[*w^+^*Sqh-GAL4]2 driver confirmed expression, although at a lower level than the reference pWIZ-ds1.688^3F^ insertion (Supplementary Fig. 1). We also validated substantial rescue of males with the severe *roX1^SMC17A^ roX2*Δ mutations upon expression of pWIZ-ds1.688^3F^, but minimal rescue by pWIZ-ds1.688^1A^ (Fig. 2b).

This raises the question of whether the disparity in biological activity is due to differences in DNA sequence, expression levels, or to differences in the size, orientation, or phasing of repeat DNA in the pWIZ transgene. To address this, we constructed transgenes with 1.688^1A^ and 1.688^3C^ sequence but with the same size, orientation, and repeat phasing as pWIZ-ds1.688^3F^ (see Materials and methods). This produced pWIZ-ds1.688^1AR^ (89% sequence identity to 1.688^3F^) and pWIZ-ds1.688^3CR^ (68% identity to 1.688^3F^) (see Supplementary Fig. 3 for sequence alignments). This strategy enables direct comparison to the original transgenes with the same repeat sequence in different phasing. To explore the role of orientation, we constructed pWIZ-ds1.688^1AR-inv^ and pWIZ-ds1.688^3CR-inv^ with the same inserts as pWIZ-ds1.688^1AR^ and pWIZ-ds1.688^3CR^ but inverted in orientation (Fig. 1b). Three independent second chromosome insertions of each new transgene were selected for further studies.

To confirm expression, total RNA was extracted from third instar male larvae carrying reconstructed transgenes and the p[*w^+^*Sqh-Gal4]2 driver (Supplementary Table 1). Primers to the *w^+^* intron, present in the unprocessed transcript, were used to assess relative expression from each insertion (Supplementary Fig. 1). All transgenes were expressed but with variability due to insertion site. Nevertheless, the range of expression levels overlaps that of the original pWIZ-ds1.688^1A^ and pWIZ-ds1.688^3F^ transgenes.

To assess the biological activity of reconstructed transgenes, expression was driven in *roX1^SMC17A^ roX2Δ* males (∼2–3% escapers) and *roX1^ex33A^ roX2Δ* males (∼25% escapers). Survival of *roX1^SMC17A^ roX2Δ* and *roX1^ex33^ roX2Δ* males expressing RNA from pWIZ-ds1.688^1AR^ and pWIZ-ds1.688^1AR-inv^ matched that achieved by the original pWIZ-ds1.688^3F^, reaching 30 and 65%, respectively (Fig. 2 and Supplementary Fig. 2; see Supplementary Tables 4 and 5 for raw data). Expression from pWIZ-ds1.688^3CR^ and pWIZ-ds1.688^3CR-inv^ also rescued males but to a somewhat lesser extent. A comparison of transgene expression and male survival reveals that the level of male rescue is largely independent of the level of RNA expression. For example, levels of ds1.688^3CR^ vary more than 3-fold among strains used in this study, but rescue of *roX1^ex33^ roX2Δ* males is indistinguishable (Supplementary Figs. 1 and 2). This suggests that expression exceeds that necessary for the maximum achievable rescue of *roX1 roX2* males by the dsRNAs produced. Taken together, these observations indicate that expression levels and differences in DNA sequence cannot account for the lack of male rescue by previously made pWIZ-ds1.688^1A^ and pWIZ-ds1.688^3C^ transgenes. Instead, the phasing of the repeat fragment introduced into pWIZ appears to determine the biological activity of transgenes that express ds1.688^X^ RNA.

**Fig. 3.**
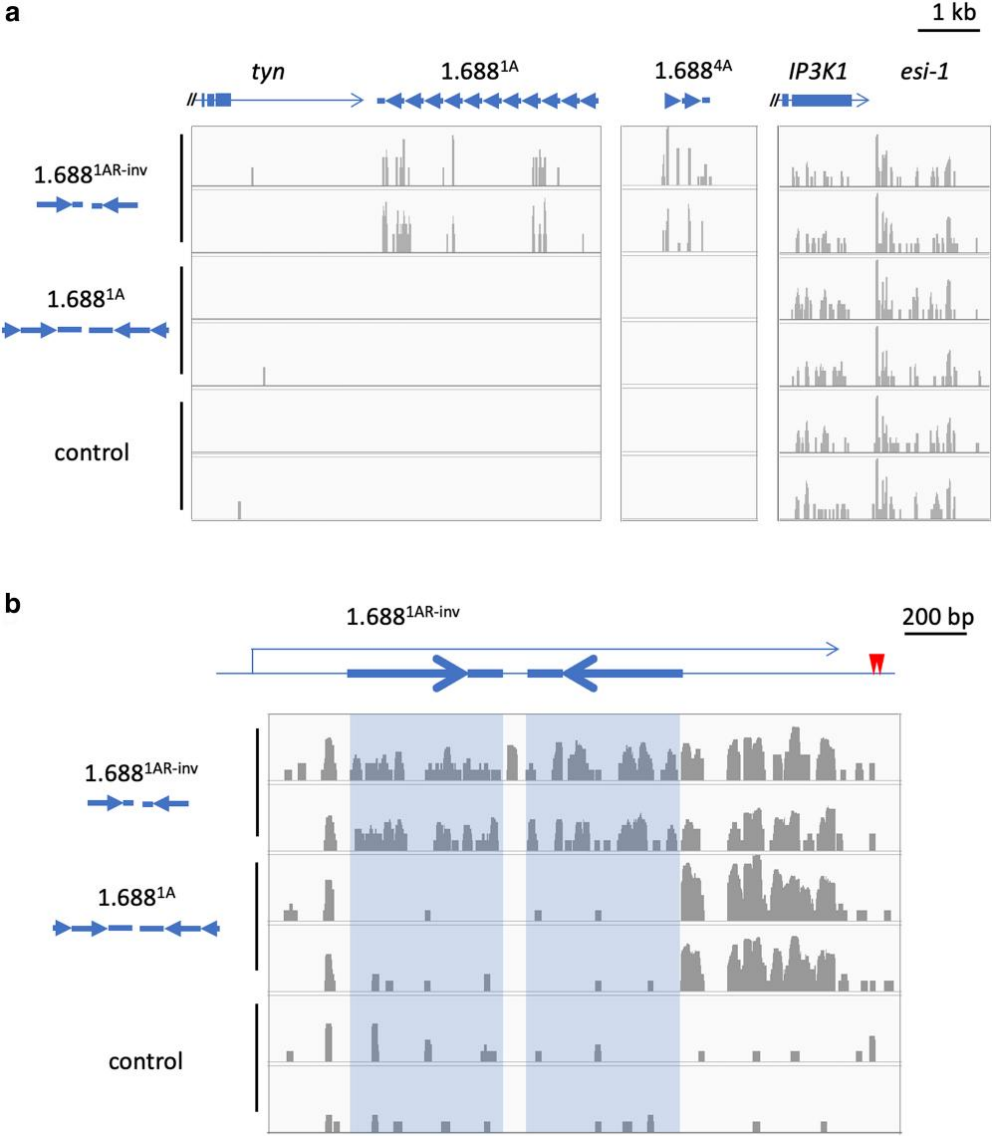
Biologically active and inactive dsRNAs differ in small RNA production. a) Alignment of small RNA from larvae carrying pWIZ-ds1.688^1AR-inv^ (top; insertion 35) pWIZ-ds1.688^1A^ (middle; insertion 2A) or no transgene (control, bottom). Two independent RNA preparations are shown for each genotype. The orientation of 1.688^1A^ sequence within pWIZ is illustrated to the left. Tandem 1.688^1A^ repeats proximal to *tyn* (left) are depicted by arrows. Related 1.688^4A^ repeats are 4.3 kb distal to CG43689 (middle). All preparations contained abundant small RNA aligning to the autosomal, hairpin RNA-producing *esi-1* locus (right). Alignments are depicted on a linear scale. b) Small RNAs were aligned to the transcript produced by pWIZ-ds1.688^1AR-inv^, schematically represented with 1.688^1A^ sequence highlighted. The SV40 terminator is upstream of two polyadenylation sites marked with red arrow heads. Alignments to the pWIZ-ds1.688^1AR-inv^ transcript are presented on a logarithmic scale. All alignments are viewed on igv genome browser (Robinson *et al*. 2011). Expression of all transgenes is driven by p[w^+^Sqh-Gal4]2.

We then asked if differences in rescue of *roX1 roX2* males reflect processing into small RNA. To assess this, we sequenced small RNA from male larvae expressing dsRNA from the original pWIZ-ds1.688^1A^, which does not rescue *roX1 roX2* males, and pWIZ-ds1.688^1AR-inv^, which achieves strong rescue. Both transgenes contain sequence from 1.688^1A^ introduced into pWIZ in the same orientation, but inserts differ in size and phasing. Small RNA from our lab reference strain (*yw*) was also sequenced. Consistent with previous studies, we detected minimal siRNA from the 1.688^1A^ region in *yw* larvae (Menon *et al*. 2014). In contrast, abundant siRNAs from pWIZ-ds1.688^1AR-inv^ aligned to 1.688^1A^ as well as 1.688^4A^ and other closely related 1.688^X^ repeats on the X chromosome (Fig. 3a). No siRNA aligning to 1.688^1A^ was detected in pWIZ-ds1.688^1A^ males. However, endogenous small RNAs from other genomic regions, such as those near the hairpin RNA-producing *esi-1* locus, were identified in all samples, demonstrating sample integrity (Fig. 3a). We then aligned all reads to the pWIZ-ds1.688^1AR-inv^ transcript, permitting no mismatches. pWIZ-ds1.688^1AR-inv^ produced abundant small RNAs aligning to both arms (Fig. 3b). However, few alignments to the insert are observed in control flies or those carrying pWIZ-ds1.688^1A^. In contrast, the SV40 terminator produces small RNAs in both transgene-carrying strains. We conclude that pWIZ-ds1.688^1A^ produces transcript but the double-stranded regions created by the 1.688^1A^ inserts are not effectively processed into siRNA. The reason for this striking disparity remains speculative.

### Expression of reconstructed transgenes enhances compensation near an autosomal 1.688^3F^ insertion

**Fig. 4.**
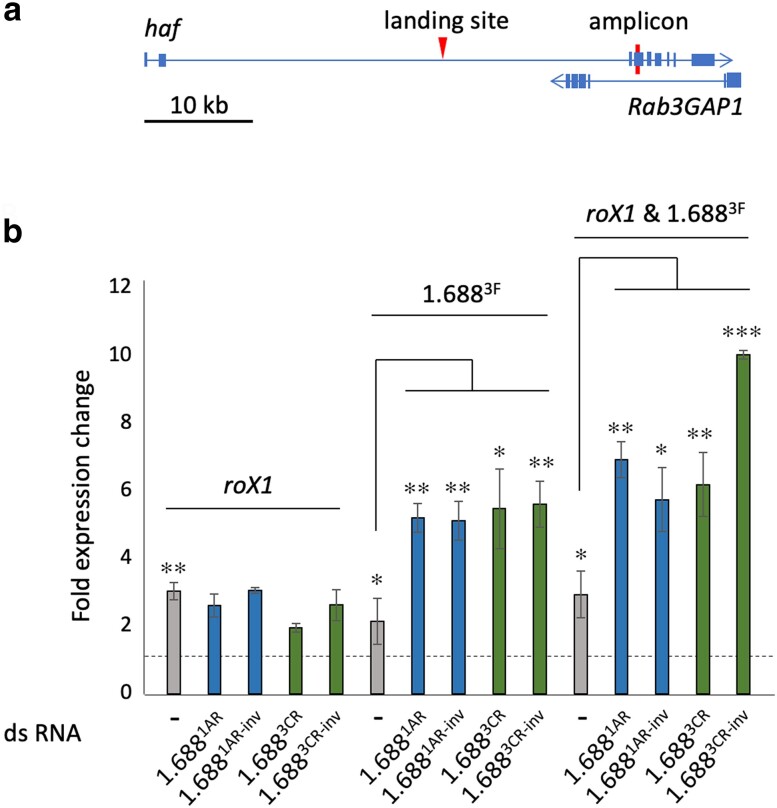
Expression of reconstructed transgenes modulates expression near an autosomal 1.688^3F^ integration. a) *roX1* and 1.688^3F^ recruiting elements are integrated in a 40 kb intron of the *haf* gene (arrow head). Primers to measure transcript accumulation amplify a *haf* exon and lie within a *Rab3GP1* intron (verticle bar). b) Accumulation of transcripts in male larvae with integrated recruiting elements *roX1,* 1.688^3F^, or both is shown by gray bars (insertions [1.688^3F^]^22A3^, [*roX1*]^22A3^, and [*roX1*+1.688^3F^]^22A3^; Joshi and Meller 2017). Additional expression of ds1.688^1AR^ or ds1.688^1AR-inv^ (blue), and ds1.688^3CR^ or ds1.688^3CR-inv^ (green) increased *haf* expression only when 1.688^3F^ was present in *haf*. Expression is normalized to *dmn* and that in lab reference *yw* males is set to 1 (line). The significance of expression with recruiting elements alone is with respect to *yw* males as indicated by asterisks. All other statistical comparisons are between males with recruiting elements alone and those with each recruiting element and dsRNA expression. Error bars represent SEM from three biological replicates. *P < 0.05; **P < 0.01, ***P < 0.001, as determined by Student’s *t* test.

We postulate that the ability of reconstructed transgenes to rescue *roX1 roX2* males involves small RNA-directed chromatin modification at hundreds of related sequences on the X chromosome. We previously demonstrated functional compensation of hemizygous autosomal genes near integrations of DNA from the 1.688^1A^, 1.688^3C^, and 1.688^3F^ repeats (Joshi and Meller 2017). Less than 2 kb of 1.688^3F^ DNA was capable of recruitment of MSL proteins detectable on polytene preparations and increased expression of active genes over 100 kb from an autosomal integration site (Deshpande and Meller 2018). The expression of nearby genes was enhanced upon production of ds1.688^3F^, revealing that small RNA from 1.688^3F^ is able to promote recruitment at related genomic sequences (Deshpande and Meller 2018). We took advantage of autosomal integrations of 1.688^3F^ and *roX1* recruiting elements in the *hattifattener* (*haf)* gene (cytological position 22A3) to determine if expression of reconstructed dsRNA-expressing transgenes also enhances recruitment (Fig. 4a).

We generated larvae that express reconstructed transgenes and have integrations of 1.688^3F^, *roX1,* or both elements in a *haf* intron ([1.688^3F^]^22A3^, [*roX1*]^22A3^, or [*roX1&*1.688^3F^]^22A3^, Supplementary Table 1). Accumulation of *haf* mRNA in male larvae was determined by qRT-PCR. Expression was normalized to *dmn* (DCTN2-p50) and expression in the reference *yw* strain set to one. Integration of 1.688^3F^, *roX1,* or both recruiting elements increased *haf* expression in males from 2- to 3-fold (gray bars, Fig. 4b). When only a *roX1* recruiting element was present, no further increase was achieved upon expression of dsRNA from reconstructed transgenes. This agrees with the idea that recruitment of dosage compensation by the CES (present in *roX1*) and the 1.688^X^ repeats occurs by different mechanisms (Joshi and Meller 2017). In contrast, *haf* expression increased 5-fold over that in the *yw* control when the 1.688^3F^ recruiting element was integrated and dsRNAs were expressed. When both 1.688^3F^ and *roX1* were present, *haf* expression increased 6- to 10-fold over that of the control in response to dsRNA production (Fig. 4b and Supplementary Table 7). In this context, we see essentially no difference in response to transgenes expressing 1.688^1A^ and 1.688^3C^ dsRNA, even though 1.688^3C^ shares only 68% identify with the 1.688^3F^ recruiting element but 1.688^1A^ shares 89%. We conclude that siRNAs from multiple 1.688^X^ repeats are capable of promoting recruitment by related genomic sequences. This suggests that sources of siRNA that participate in X recognition are likely to be highly redundant. The levels of *haf* activation achieved are considerably higher than the 2-fold increase anticipated for full dosage compensation. This has been previously noted for autosomal *roX1* insertions and is attributed to disruption of chromatin-based silencing upon recruitment of the MSL complex (Kelley and Kuroda 2003).

### Pericentromeric 359 bp satellites contribute to X recognition

**Fig. 5.**
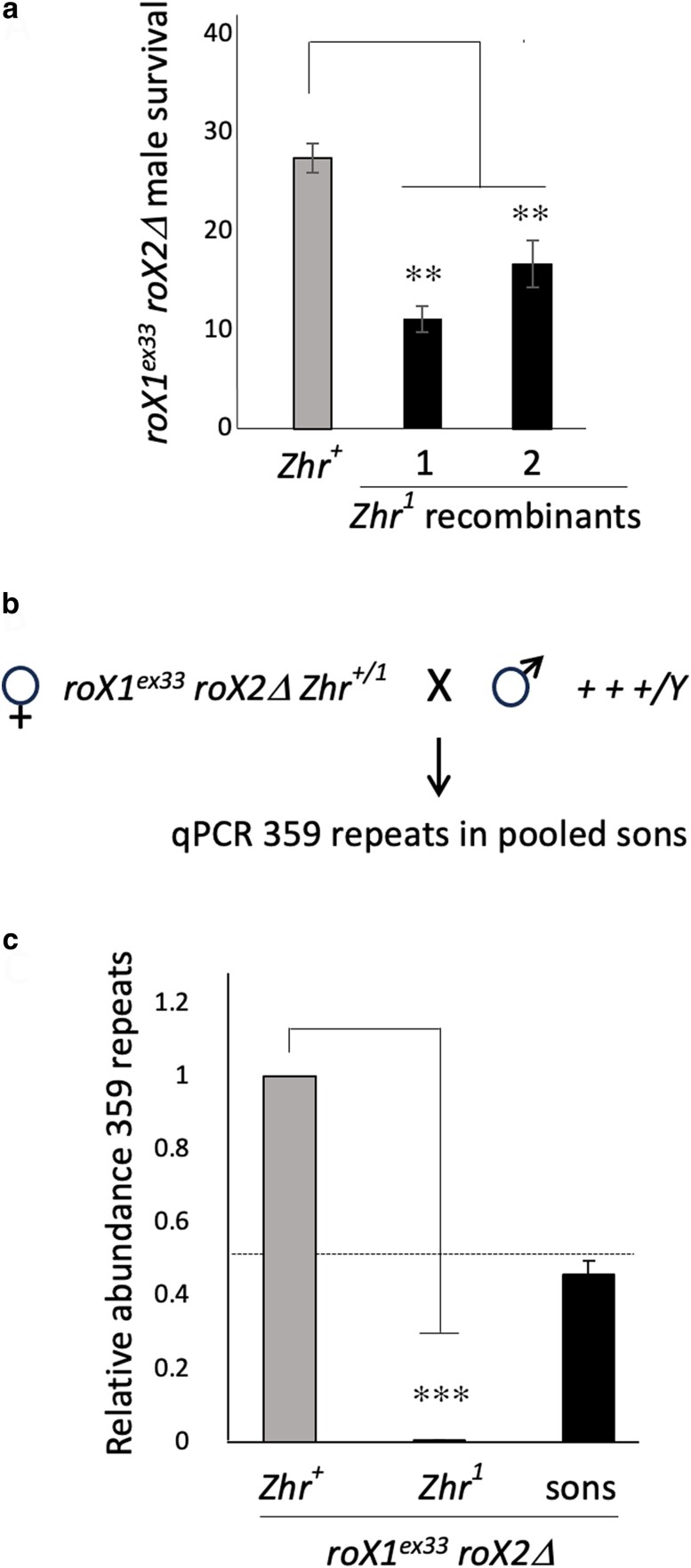
Pericentromeric 359 bp satellites influence dosage compensation. a) Eclosion of *roX1^ex33^ roX2*Δ males that are wild-type for *Zhr* (*Zhr^+^*) and two independent *roX1^ex33^ roX2*Δ *Zhr^1^* recombinants. Error bars represent SEM of four biological replicates. b) *roX1^ex33^ roX2*Δ *Zhr^1^*^/+^ mothers are mated to reference *yw* males (+++/Y) and male offspring pooled for DNA extraction and qPCR of 359 bp repeats. c) Abundance of 359 bp repeats in *roX1^ex33^ roX2*Δ *Zhr^+^* (left) is set to 1. *Zhr^1^* (center) lacks the 359 bp repeat array (see Supplementary Table 2 for primers). Pooled sons from *roX1^ex33^ roX2*Δ *Zhr^1^*^/+^ mothers (right) reveal approximately equal numbers of *Zhr^1^* and *Zhr^+^* sons. Error bars represent SEM from four biological replicates. **P < 0.01, ***P < 0.001, as determined by Students *t* test.

Over 10 Mb of proximal X heterochromatin is composed of closely related 359 bp satellite repeats (Hsieh and Brutlag 1979; Lohe *et al.* 1993). The pericentromeric repeats are a potential source of siRNAs that participate in dosage compensation. To test this, we recombined the *Zygotic hybrid Rescue* (*Zhr^1^*) mutation, deleted for the pericentromeric 359 bp repeats, with *roX1^ex33^ roX2*Δ (Sawamura and Yamamoto 1993). Recombinants were validated by qPCR of the 359 repeats. *roX1^ex33^ roX2*Δ *Zhr^1^* and *roX1^ex33^ roX2Δ Zhr^+^* virgins were mated to males from our *yw* reference strain and the eclosion of males from each mating was calculated based on female survival. Control r*oX1^ex33^ roX2*Δ *Zhr^+^* sons eclosed at 27% (Fig. 5a and Supplementary Table 6). However, *roX1^ex33^ roX2*Δ *Zhr^1^* sons were recovered at 10–15%, suggesting that the pericentric 359 bp repeats also contribute to compensation.

*Zhr^+^* produces a maternally transmitted factor, presumably small RNA, that is necessary for assembly of the 359 bp repeats into heterochromatin in the zygote (Yuan and O’Farrell 2016; Ferree and Barbash 2009). This raised the possibility that maternal deposition of small RNA from the 359 bp repeats might also influence dosage compensation. To determine if this effect was maternal or zygotic, we mated *roX1^ex33^ roX2*Δ females heterozygous for *Zhr^1^* (*roX1^ex33^ roX2*Δ *Zhr^1/+^)* to *yw* males. We reasoned that if the benefit of wild-type *Zhr^+^* was zygotic, most eclosing male offspring would carry this allele. However, if *Zhr^+^* confers a maternal effect, equal numbers of *Zhr^1^* and *Zhr^+^* sons are expected. To estimate the fraction of sons with each allele, male offspring were pooled for DNA extraction and the abundance of 359 bp repeats determined by qPCR (Fig. 5b). Amplification reveals approximately equal recovery of each class of sons (Fig. 5c). Lack of enrichment for *Zhr^+^* in male offspring suggests that the 359 bp repeats act maternally to support the survival of *roX1 roX2* sons.

## Discussion

Recognition of X-linked genes is essential for dosage compensation, but how this is accomplished is not fully understood in any system. At least two classes of recruiting elements, the CES and 1.688^X^ satellites, act to attract the compensation machinery the *Drosophila* X chromosome but do so by distinct mechanisms. For example, CLAMP binds to and is necessary for recruitment by the CES, but loss of CLAMP does not reduce recruitment by 1.688^X^ repeats (Makki and Meller 2024). Conversely, genes in the siRNA pathway support recruitment by 1.688^X^ repeats but are dispensable for the CES. We postulate that cooperation between multiple classes of recruiting elements achieves faithful recognition of the X chromosome by the MSL complex. Both CES and 1.688^X^ repeats engage in long-range interactions, suggesting that organization of the X chromosome may contribute to selective identification (Ramírez *et al.* 2015; Sproul *et al.* 2020).

The involvement of the siRNA pathway in X recognition, abundance of 1.688^X^ repeats on the X chromosome, and rescue of *roX1 roX2* males by expression from the pWIZ-ds1.688^3F^ transgene, suggested the involvement of an siRNA-directed chromatin modification system (Menon and Meller 2012; Menon *et al*. 2014). Mutation of a single copy of genes involved in production of siRNA and chromatin modification, including *Dicer2, Ago2,* and *Su(var)3-9*, enhance the male lethality of *roX1 roX2* mutations supporting this idea (Menon and Meller 2012; Deshpande and Meller 2018). In accord with this, histone 3 di-methylated on lysine 9 (H3K9me2) is enriched on 1.688^X^ repeats, and this mark is enhanced around an autosomal 1.688^3F^ insertion upon expression of ds1.688^3F^ RNA (Deshpande and Meller 2018). Recruitment of the MSL complex and upregulation of autosomal genes near insertions of 1.688^1A^ or 1.688^3C^ DNA is also enhanced by ds1.688^3F^ RNA, suggesting that 1.688^X^ repeats function redundantly to mark the X (Joshi and Meller 2017). Although these observations implicated 1.688^X^ siRNA in X recognition, the fact that expression from the original pWIZ-ds1.688^1A^ and pWIZ-ds1.688^3C^ transgenes failed to rescue *roX1 roX2* males raised questions regarding the origin of the siRNAs (Menon *et al.* 2014). The proximity of 1.688^3F^ and *roX1* is intriguing and suggests that 1.688^3F^ might have a unique function. However, we have been unable to find evidence to support this. *roX1 roX2* mutants carrying *roX1^VM18A^*, a deletion of the essential 3′ end of *roX1,* the entire 1.688^3F^ repeat and part of *echinus*, do not exhibit more complete male lethality than other severe *roX1* mutants that retain the 1.688^3F^ repeats (Menon *et al*. 2014). Transcripts spanning *roX1* and 1.688^3F^ are depicted in the *Drosophila* genome annotation, but we have been unable to detect evidence of these longer transcripts in embryos or adults and suspect that annotation is based on rare read through transcripts. Nor have we discovered that the proximity of the 1.688^3F^ repeats and *roX1* is important to their function as each is capable of acting independently to recruit compensation to nearby genes (Joshi and Meller 2017). Demonstration of *roX1 roX2* male rescue by reconstructed transgenes that express double-stranded 1.688^1A^ and 1.688^3C^ RNAs, and evidence that the pericentromeric 359 bp repeats also contribute to dosage compensation, suggests high redundancy in the sources of small RNAs that support X recognition.

The striking difference in biological activity between the original ds1.688^3F^, ds1.688^1A^, and ds1.688^3C^ transgenes now appears due to an inability of cells to process dsRNA from the original transgenes. As phasing of the repeats in the dsRNA was identified as most likely responsible for this, it is possible that dsRNAs with some phasings adopt a conformation that impedes Dicer processing. Dicer preferentially processes substrates based on kinetic and thermodynamic characteristics and sequence composition may also influence Dicer cleavage (Vermeulen *et al*. 2005; Lee *et al.* 2023). Modeling of dsRNA secondary structures failed to reveal large differences between the most energetically favorable conformations of ds1.688^1A^ and ds1.688^1AR-inv^. Ineffective RNAi has also been linked to degradation of dsRNA by endogenous nucleases (Singh *et al.* 2017). Understanding why siRNA production from the original pWIZ-1.688^1A^ and pWIZ-1.688^3C^ transgenes fails will require further studies to explore these ideas. We also acknowledge that the phasing in the original pWIZ-1.688^3F^ transgene may not be optimal for small RNA production, leaving open the possibility that even higher activity might be obtained by trial and error. But the current studies do reveal that the 1.688^3F^ satellite is not uniquely able to produce siRNA that supports X recognition as this property is shared by other 1.688^X^ repeats and likely also the pericentric 359 bp satellites.

Female hybrids generated by mating *D*. *melanogaster* males to *D. simulans* females, a species lacking the large block of 359 bp repeats on the X chromosome, fail to assemble heterochromatin over the 359 bp repeats and experience mitotic catastrophe when the *D. melanogaster* X chromosome fails to segregate (Ferree and Barbash 2009; Yuan and O'Farrell 2016). The maternal factor present in *D. melanogaster* eggs is suspected to be small RNA from the 359 bp repeats. *Zhr^1^* lacks 359 bp repeats, thus rescuing mitosis in the daughters of *D. melanogaster Zhr^1^* males mated to *D. simulans* females (Sawamura and Yamamoto 1993). Our studies suggest that the pericentric 359 bp repeats also play a maternal role in identification of the X chromosome for dosage compensation. Interestingly, a strong modifier of *roX1 roX2* male lethality was previously mapped to the proximal X (Stuckenholz *et al.* 2003). Our findings suggest that this modifier could be an allelic variant of *Zhr*. The observation that small RNAs aligning to 1.688^X^ satellites are abundant in early embryos but absent from wild-type larvae raises the question of when these small RNAs are produced (Menon *et al.* 2014). Many euchromatic 1.688^X^ repeats are transcribed in male larvae but small RNAs aligning to these repeats are rare in this stage (Menon *et al*. 2014). Heterochromatic satellites are transcribed in the female germ line and processed into small RNA (Wei *et al.* 2021). It is possible that the primary source of the small RNAs active in dosage compensation are maternal. This is consistent with a role for satellite repeats and siRNA in identification of X chromatin during the establishment of dosage compensation in the zygote.

The X chromosomes of many Drosophilids are enriched with chromosome-specific repetitive sequences. These are up to 50 times more abundant than autosome-specific repeats in some species (Gallach 2014). Strikingly, the neo-X chromosome in *D. pseudoobscura* (arm XR) shares this enrichment, suggesting that acquisition of satellite repeats is an early step in the evolution of differentiated sex chromosomes. Repetitive DNA, marked by turnover in sequence and copy number, is implicated in the evolution of sex chromosomes (Smith 1976; Charlesworth *et al.* 1994; Bayes and Malik 2009; Gallach 2014). Theoretical models of sex chromosome evolution usually focus on accumulation of mutations on the Y chromosome, erosion of coding potential and the subsequent need to increase expression of hemizygous X-linked genes in males (Charlesworth 1996). Both the CES and X chromosome-specific satellite repeats rapidly populate neo-X chromosomes created by sex chromosome and autosomal fusions (Alekseyenko *et al.* 2013; Ellison and Bachtrog 2013; Gallach 2014). Given the power of CES and satellite repeats to attract compensation to nearby genes, it is possible that the acquisition of compensation precedes the loss of some Y-linked homologs. This could produce over expression that would exert a selective pressure promoting loss of the Y-linked homolog, accelerating sex chromosome differentiation.

## Supplementary Material

iyae074_Supplementary_Data

## Data Availability

Strains and plasmids are available upon request. The authors affirm that all data necessary for confirming the conclusions of the article are present within the article, figures, and tables. Sequence data can be downloaded from http://www.ncbi.nlm.nih.gov/bioproject/1050379 with accession PRJNA1050379. Supplemental material available at GENETICS online.
