# Supplementary material for: siRNA that participates in Drosophila dosage compensation is produced by many 1.688X and 359 bp repeats
Source: Genetics. 2024 May 8;227(3):iyae074. doi: 10.1093/genetics/iyae074 (PMC11228850; doi:10.1093/genetics/iyae074)
Supplement: iyae074_Supplementary_Data [file iyae074_supplementary_data.pdf]

Supplemental materials

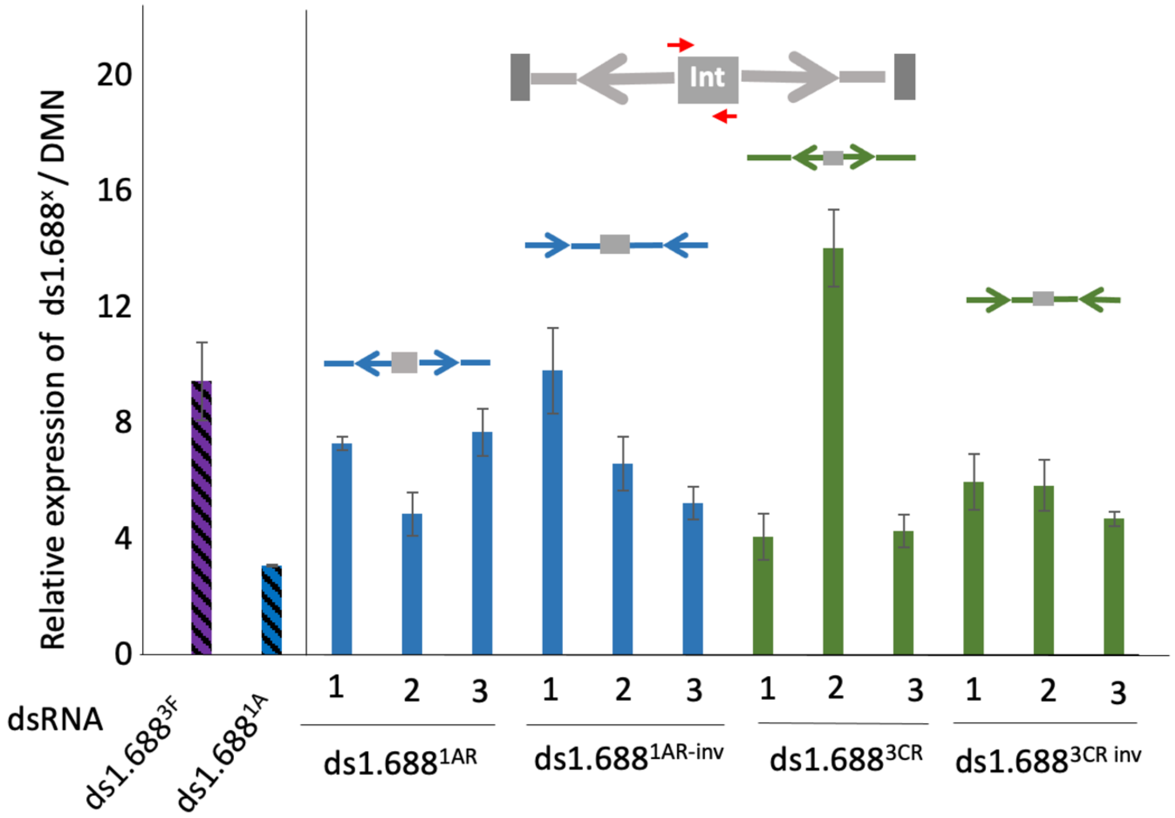

Figure S1 Accumulation of dsRNA in male third instar larvae. (A) Left - insertions of pWIZ-ds1.688^{3F} and pWIZ-ds1.688^{1A} from Menon et al., 2014. Right - reconstructed pWIZ-ds1.688^{XR} insertions described in this manuscript. All expression is driven by p[w^{+}Sqh-Gal4]2. Primers amplify the white (w) intron, situated between the pWIZ cloning sites. Expression is normalized to *dmn*. Error bars represent the SEM of three biological replicates.

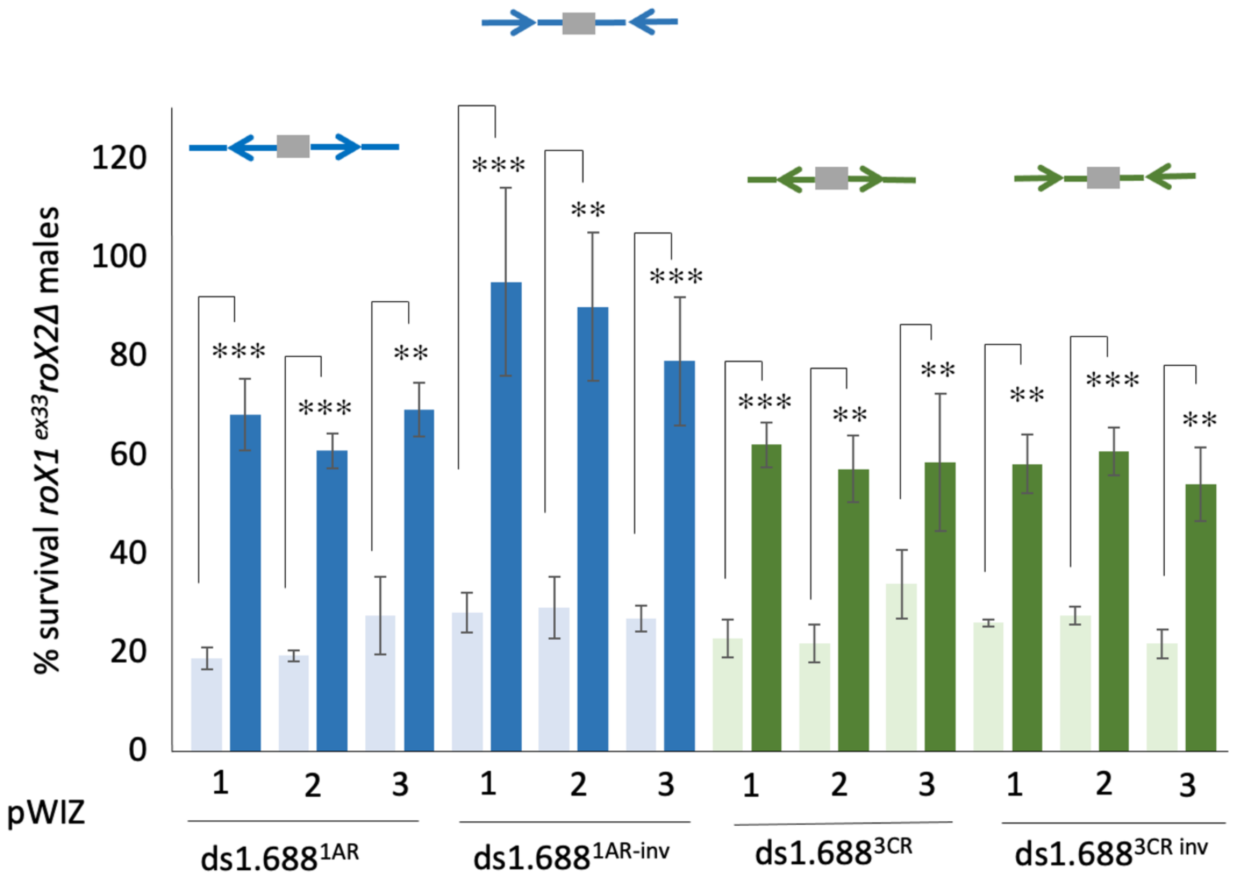

Figure S2 Expression from pWIZ-ds1.688^{XR} transgenes rescues *roX1^{ex33} roX2Δ* males. Three independent insertions of each pWIZ transgene were mated to *roX1^{ex33} roX2Δ;* p[w^{+}Sqh-Gal4]2/+ females. Male eclosion is calculated based on total female offspring (Table S4). Error bars represent SEM of four biological replicates. *P < 0.05; **P < 0.01, ***P < 0.001, as determined by Students t test.

Figure S3 Alignment of insert 1.688^{X} sequences. 1.688^{3F}, 1.699^{1A} and 1.688^{3C} are from Menon *et al.*, (2014). 1.688^{1AR} and 1.688^{3CR} are the inserts in reconstructed transgenes described in this study. Arrow indicates the orientation of repeats. *Eco*RI (red) or *Sac*I (green) are arbitrarily assigned as the boundary between tandem repeats.

```
1.6883F      accaagttatggaaaaaccccgtttgtaaatatcaactttttggcaattcatattttgt   60
1.6881A      -------------gtttacttatttacttttggcaatcataaaaacacatatagggagag   47
1.6881AR     gtcaggttatagagaaaaccccgtttgtaaatatccgattttggcaattcatattttgt   60
1.6883C      ------------------------------------------------------------   0
1.6883CR     -cgatgttatggcgaaaataccgttagtaaatcacgtttt-tggcaaaagccgtttttcc   58

1.6883F      ttatttttggttaaaaagaatcagtatttctcaatagcataaaaaatatttgtccaaaa   120
1.6881A      ttatttttgcctagaaagaatctgtactttctcaatagcattaaaaataactgtccaaga   107
1.6881AR     ttatttttgcctagaaagaatctgtactttctcaatagcattaaaaatatttgtccaaaa   120
1.6883C      ------------------------------------------------------------   0
1.6883CR     aaaattcggtcataaaaaaatccgtttttggcgacaattttaaaataattgcctgaat   118

1.6883F      gtgaaatgccatacctcgttgaattcgtaacaaaatttcctatcgacctgtattcagaaa   180
1.6881A      gcgaaatggcatacctcgttgaattcgtaacaaaattccccatcgacctgtattcagaaa   167
1.6881AR     gcgaaatggcatacctcgttgaattcgtaacaaaattccccatcgacctgtattcagaaa   180
1.6883C      -------------------------------------cccaatccaactgtaaccccgaa   23
1.6883CR     atggaatgtcataccgcgttgagctcgtaataaaattcccaatccaaccgtaaccccgaa   178
                                                   ** *** * * ***  *   **

1.6883F      tggaaagtcaaattttttgcaatttttcgcaaa--ttttaatgaaaa----attttatca   234
1.6881A      tggaaattcaattttttttgccattttcgcaaa--tttttatgatggtaacccttatcga   225
1.6881AR     tggaaattcaattttttttgccattttcgcaaa--tttttatgatggtaacccttatcga   238
1.6883C      ttggaattctatttttttagccattttttga--aaattttcatgatgttaccccttacaaa   81
1.6883CR     ttggaattctatttttttagccattttttttttgaaattttcatg-atgttacccctacaaa   237
             * * ** ** * ***** ** ******     *  **** ***           *    *

1.6883F      aaaatgcgaaaatttgttaaaattttattttgatatttataaaaatagtgatagggat   294
1.6881A      aaaatgtgaaaatgtgttaaaattttattttgatgataataaaaatagtgatacggat   285
1.6881AR     aaaatgtgaaaatgtgttaaaattttattttgatgataataaaaatagtgatacggat   298
1.6883C      aaatgcgaaaattgacccgaaaaattaatttccctaaatccttccaaaagtgatagggat   141
1.6883CR     aaatgcgaaaattgacccgaaaaattaatttccctaaatccttccaaaagtgatagggat   297
             ***     *** *      ***  ** ****       *  *   ** ******* ****

1.6883F      agttaggtatgtttattggcagtataaaacagtcttcatttaagcggtaatatcttttt   354
1.6881A      agttcgctatgtttattggcagtacaaaacagtcttcatttaaacggttatatcttttt   345
1.6881AR     agttcgctatgtttattggcagtataaaacagtcttcatttaaacggttatatcttttt   358
1.6883C      cgttagcactgctaaatagctgctcaaagcagttattctttcatctatatgacattttt   201
1.6883CR     cgttagcactgctaaatagctgctcaaagcagttattctttcatctatatgacattttt   357
              *** *   ** * * * ** *   *** ****  *  *** * *  *   *  ******

1.6883F      -ggccaccttatagagataaccccgtttgtaaatatcaaattttggcaattcttatttt   413
1.6881A      -ggtcaggttatagagaaaaccccgtttgtaaatatccgattttggccattttatttt   404
1.6881AR     -ggtcaggttatagagaaaaccccgtttgtaaatatccgattttggcaattcttatttt   417
1.6883C      agcg-atgttatggcgaaaataccgttagtaaaatcacgttttgacaaaacccgatttt   260
1.6883CR     tagcgatgttatggcgaaaataccgtta-gtaaatcacgttttgacaaaacccgatttt   416
                  *  **** * ** **  *****    **       ****     *     *****
1.6883F      gtttattttggttaaaaagaatcagtatttctcaatagcataaaaaatagttgaccaa   473
1.6881A      gtttatttcggtaaa---aaagactgtatttctcaatagcattaaaaataactgtccaa   461
1.6881AR     gtttatttcggtaaa---aaagactgtatttctcaatagcattaaaaataactgtccaa   474
1.6883C      ccaaaattcggtcat-aaaaaatccgtttttcggcacaactttaaaaataatt-gctga   318
1.6883CR     ccaaaattcggtcataaaaaaatccgtttttcggcacaactttaaaaataatt-gctga   475
                 * **  *       * *  * ** *****   * * * * *******  *  *  *

1.6883F      aagggaaatgccatacctcgtt--------------------------------------   495
1.6881A      gagcgaaatggcatacctcgttgaattcgtaacaaaattccctatcgactgtattcagaa   521
1.6881AR     gagcgaaatggcatacctcgtt--------------------------------------   496
1.6883C      atgtggaatgtcataccgcgttgagctcgtattaaaatttccaatcaaactgtgcaaaaa   378
1.6883CR     atgtggaatgtcataccgcgtt--------------------------------------   497
               * * **** ****** ****
```

```
1.6883F     ------------------------------------------------------------     495
1.6881A     atgaaaattcagatttttgccatttttcgcaaattttaatgatggtaccccttatcaag     581
1.6881AR    ------------------------------------------------------------     496
1.6883C     tatagagttttatttttcgtcagttttgaaattttccccttaaaaaaaaaaatgcaa     438
1.6883CR    ------------------------------------------------------------     497

1.6883F     ------------------------------------------------------------     495
1.6881A     aatgcg---aaaacgtgttt----------------------------------------     598
1.6881AR    ------------------------------------------------------------     496
1.6883C     aaacttatcaaaaattatttttgtttttgaatccgaaaaattgtaataaagatcgttagc     498
1.6883CR    ------------------------------------------------------------     497

1.6883F     ------------------------------------------------------------     495
1.6881A     ------------------------------------------------------------     598
1.6881AR    ------------------------------------------------------------     496
1.6883C     agtagttataagcttggcaaaactattactcttttagccatatgaccatttttggccaat     558
1.6883CR    ------------------------------------------------------------     497

1.6883F     ------------------------------------------------------------     495
1.6881A     ------------------------------------------------------------     598
1.6881AR    ------------------------------------------------------------     496
1.6883C     atgtgaagaaaaagcattcgtgaatattaaatttttgcgtaaagtcgttctaagttcttt     618
1.6883CR    ------------------------------------------------------------     497

1.6883F     ------------------------------------------------------------     495
1.6881A     ------------------------------------------------------------     598
1.6881AR    ------------------------------------------------------------     496
1.6883C     gctaatattatgattaagttatttcttcaagtgttgcttaggtcgtctcgctctaacgct     678
1.6883CR    ------------------------------------------------------------     497

1.6883F     ------------------------------------------------------------     495
1.6881A     ------------------------------------------------------------     598
1.6881AR    ------------------------------------------------------------     496
1.6883C     ggcagagatattagtgacatgcagcttgcagtttttaaaatgccggaaagtggggaaaag     738
1.6883CR    ------------------------------------------------------------     497

1.6883F     ------------------------------------------------------------     495
1.6881A     ------------------------------------------------------------     598
1.6881AR    ------------------------------------------------------------     496
1.6883C     tgagtgggcggtggacggggcgaggtgaaaggaaaggaaggacgcttcaaggatgctgcg     798
1.6883CR    ------------------------------------------------------------     497

1.6883F     --------------     495
1.6881A     --------------     598
1.6881AR    --------------     496
1.6883C     gttttgttcttgtc     812
1.6883CR    --------------     497
```

Table S1 *Drosophila* strains used in this study.

Description	Genotype	Flybase Id # Citations
p[w^{+}Sqh-Gal4]2	P{w^{+mC}sqh-Gal4}2	FBti0074562
Zhr^{1}	*Zhr^{1}*	25140 (BDSC) FBst0025140
pWIZ-ds1.688^{1AR}	pWIZ-ds1.688^{1AR} 7C (1)	This study (Figure 2)
	pWIZ-ds1.688^{1AR} 7B (2)	
	pWIZ-ds1.688^{1AR} 5G (3)	
pWIZ-ds1.688$^{1AR-inv}$	pWIZ-ds1.688$^{1AR-inv}$ 17B (1)	This study (Figure 2)
	pWIZ-ds1.688$^{1AR-inv}$ 10 (2)	
	pWIZ-ds1.688$^{1AR-inv}$ 35 (3)	
pWIZ-ds1.688^{3CR}	pWIZ-ds1.688^{3CR} 9H (1)	This study (Figure 2)
	pWIZ-ds1.688^{3CR} 20A (2)	
	pWIZ-ds1.688^{3CR} A13B (3)	
pWIZ-ds1.688$^{3CR-inv}$	pWIZ-ds1.688$^{3CR-inv}$ B7A (1)	This study (Figure 2)
	pWIZ-ds1.688$^{3CR-inv}$ B12A (2)	
	pWIZ-ds1.688$^{3CR-inv}$ B1D (3)	
roX1 roX2	*yw roX1^{ex33} roX2*∆	Deng *et al.* 2005
	w roX1^{SMC17A}roX2∆	Apte *et al.* 2014
[*roX1*+1.688^{3F}]^{22A3}	PBac{*y^{+} roX1 w^{+mc}* 1.688^{3F}} VK00037	Joshi & Meller 2017
[1.688^{3F}]^{22A3}	PBac{*y^{+}* 1.688^{3F}} VK00037	
[*roX1*]^{22A3}	PBac{*y^{+} roX1*} VK00037	
pWIZ-ds1.688^{1A}	*yw*; pWIZ-hp1.688^{1A} 2A	Menon *et al.* 2014
pWIZ-ds1.688^{3C}	*yw*; pWIZ-hp1.688^{3C} 50A	
pWIZ-ds1.688^{3F}	*yw*; pWIZ-hp1.688^{3F} 12	

Table S2 Primers used in construction and validation.

Name	Sequence
Hsp70F2	GCAACTACTGAAATCTGCCAAG
SV40R2	ATATGGATCCGGTACCTCGAGAACCCCTCCCACACCTC
Int2R	AGGGTCCAATTACCAATTG
Int2L	GACTGCGAATAGAAACTCAC
mJOF	TGCATGATCTACGTGCGTCACATGC
mJOR	CAACAGCGGCTACTGAATCTGAGC
1.6881A-(F1)	GTCAGGTTATAGAGAAA
1.6881A-(R2)	TCGGATATTTACAAACGGG
1.688-1A-(F2)	AGTGATACGGATAGTTCGCTATGT
3C_F	GCTCGAGACTAGTCGATGTTATGGCGAAAATACCGTTAGTAAAATCACGTTTTTGG
3C_R	GCGCCGGCCGCTCTAGAACGCGTATGACATTCCAC
PWIZ-3C(F)	CCCAATCCAACTGTAAACCCGAA
3C_cen_R	CCAATTCGGGGTTACAGTTGGATTGGG
3C_Fo	GCTCGAGACTAGTCGATGTTATGG
3C_Ro	GCGCCGGCCGCTCTAGAAACGCGGT
T7	TAATACGACTCACTATAGGG

Table S3 Primers used for qRT PCR and qPCR

Region	Primer name	Sequence	Working Conc. (nM)	% Efficiency
pWIZ intron	SB_intron F3	CGCAGTCGGCTGATCTGTGTG	300	110.1
	SB_intron R4	CTGAGTTTCAAATTGGTAATTGGAC		
1.688^{1A}	RNR1-1A	TATTTACAAACGGGGTTTTCTCTATAACCTG	150	108.5
	RNR3-1A	CGTAACAAAATTCCCCATCGACCTG		
1.688^{3C}	RNR3C F1	TTCAGCAATTATTTTTAAAGTTGTGCCG	150	100
	RNR3C R1	CTTACAAAAAATGCGAAAAATTGACCCGA		
haf	Hap F2	AGCTGAACTGCTGGATT	300	95.6
	Hap R2	AGGGTGGACAGCTTTGTTAC		
359 satellite	359 F2	TTGTCTGAATATGGATGTTCATATCTC	150	104.6
	359 R3	TTCGTTATAACTTGGCTAAAAATGG		
dmn	Dmn_F	GACAAGTTGAGCCGCCTTAC	300	98.5
	Dmn_R	CTTGGTGCTTAGATGACGCA		

	Control		dsRNA expression	
**RNA-expressing transgene**	**Females**	**Males**	**Females**	**Males**
pWIZ-ds1.688^{3F} (Menon et al. 2014)	988	14	1058	327
pWIZ-ds1.688^{1A} (Menon et al. 2014)	942	9	800	32
pWIZ-ds1.688^{1AR} 1	1013	5	1129	260
pWIZ-ds1.688^{1AR} 2	888	17	876	121
pWIZ-ds1.688^{1AR} 3	1000	19	979	251
pWIZ-ds1.688$^{1AR\text{-}inv}$ 1	642	17	722	301
pWIZ-ds1.688$^{1AR\text{-}inv}$ 2	692	9	680	229
pWIZ-ds1.688$^{1AR\text{-}inv}$ 3	803	14	822	304
pWIZ-ds1.688^{3CR} 1	492	5	517	125
pWIZ-ds1.688^{3CR} 2	593	7	647	128
pWIZ-ds1.688^{3CR} 3	424	8	436	39
pWIZ-ds1.688$^{1AR\text{-}inv}$ 1	515	4	541	59
pWIZ-ds1.688$^{1AR\text{-}inv}$ 2	387	6	431	62
pWIZ-ds1.688$^{1AR\text{-}inv}$ 3	416	7	451	69

Table S4 *roX^{SMC17A} roX2Δ* male survival. Males with dsRNA-expressing transgenes were mated to *roX1^{SMC17A} roX2Δ;* p[w$^+$Sqh-Gal4]2/+ females. Control offspring lack p[w$^+$Sqh-Gal4]2. Total female recovery was used to calculate male survival.

	Control		dsRNA expression	
**RNA-expressing transgene**	**Females**	**Males**	**Females**	**Males**
pWIZ-ds1.688^{1AR} 1	789	143	737	520
pWIZ-ds1.688^{1AR} 2	632	119	605	376
pWIZ-ds1.688^{1AR} 3	688	188	687	476
pWIZ-ds1.688$^{1AR-inv}$ 1	661	284	746	600
pWIZ-ds1.688$^{1AR-inv}$ 2	623	238	623	558
pWIZ-ds1.688$^{1AR-inv}$ 3	829	305	824	693
pWIZ-ds1.688^{3CR} 1	816	192	870	523
pWIZ-ds1.688^{3CR} 2	804	182	871	478
pWIZ-ds1.688^{3CR} 3	594	206	624	356
pWIZ-ds1.688$^{1AR-inv}$ 1	750	196	761	439
pWIZ-ds1.688$^{1AR-inv}$ 2	714	194	704	430
pWIZ-ds1.688$^{1AR-inv}$ 3	613	140	679	349

Table S5 *roX^{ex33} roX2Δ* male survival**.** Males with dsRNA-expressing transgenes were mated to *roX1^{ex33} roX2Δ;* p[w$^+$Sqh-Gal4]2/+ females. Control offspring lack p[w$^+$Sqh-Gal4]2. Total female recovery was used to calculate male survival.

X chromosome	Replicate	Female	Males
yw rox1^{ex33} rox2 Zhr^{+} (Control)	1	307	100
	2	130	47
	3	78	36
	4	209	78
yw rox1^{ex33} rox2 Zhr^{1} recombinant 1	1	197	25
	2	219	60
	3	99	25
	4	233	40
yw rox1^{ex33} rox2 Zhr^{1} recombinant 2	1	86	7
	2	145	21
	3	232	33
	4	88	8

Table S6 *Zhr* influences *roX1 roX2* male survival. Control offspring are from a mating of *yw roX1^{ex33} roX*2Δ *Zhr^{+}* females to *yw* males. Females from two independent *yw roX1^{ex33} roX*2Δ *Zhr^{1}* recombinants were mated to *yw* males and offspring recorded.

Recruiting element	dsRNA	***haf* fold increase**
roX1	-	3.028723615
roX1	ds1.688^{1AR}	2.597480721
roX1	ds1.688$^{1AR\text{-}inv}$	2.956275537
roX1	ds1.688^{3CR}	2.21823541
roX1	ds1.688$^{3CR\text{-}inv}$	2.61408864
1.688^{3F}	-	2.139199204
1.688^{3F}	ds1.688^{1AR}	5.189164597
1.688^{3F}	ds1.688$^{1AR\text{-}inv}$	5.109044115
1.688^{3F}	ds1.688^{3CR}	5.460872294
1.688^{3F}	ds1.688$^{3CR\text{-}inv}$	5.606992091
roX1 1.688^{3F}	-	2.929235864
roX1 1.688^{3F}	ds1.688^{1AR}	6.911825171
roX1 1.688^{3F}	ds1.688$^{1AR\text{-}inv}$	5.732579992
roX1 1.688^{3F}	ds1.688^{3CR}	6.180494713
roX1 1.688^{3F}	ds1.688$^{3CR\text{-}inv}$	10.00998459

Table S7 Increase of *haf* expression upon ds1.688^{X} RNA expression. *haf* expression is expressed as fold change over that in control *yw* male larvae. Genotypes of integrations in *haf* are: PBac{y^{+} *roX1*} VK00037 (*roX1*); PBac{y^{+} 1.688^{3F}} VK00037 (1.688^{3F}) and PBac{y^{+} *roX1* w^{+mC} 1.688^{3F}} VK00037 (*roX1*&1.688^{3F}).

Supplemental References

Apte, M. S., V. A. Moran, D. U. Menon, B. P. Rattner, K. H. Barry *et al.*, 2014 Generation of a useful roX1 allele by targeted gene conversion. G3 (Bethesda) 4**:** 155-162.10.1534/g3.113.008508 https://doi.org/10.1534/g3.113.008508

Deng, X., B. P. Rattner, S. Souter and V. H. Meller, 2005 The severity of roX1 mutations is predicted by MSL localization on the X chromosome. Mech Dev 122**:** 1094-1105. https://doi.org/10.1016/j.mod.2005.06.004

Joshi, S. S., and V. H. Meller, 2017 Satellite Repeats Identify X Chromatin for Dosage Compensation in Drosophila melanogaster Males. Curr Biol 27**:** 1393-1402.e1392. https://doi.org/10.1016/j.cub.2017.03.078

Menon, D. U., C. Coarfa, W. Xiao, P. H. Gunaratne and V. H. Meller, 2014 siRNAs from an X-linked satellite repeat promote X-chromosome recognition in Drosophila melanogaster. Proc Natl Acad Sci U S A 111**:** 16460-16465. https://doi.org/10.1073/pnas.1410534111
